# Physiological β‐amyloid clearance by the liver and its therapeutic potential for Alzheimer’s disease

**DOI:** 10.1002/alz.088709

**Published:** 2025-01-03

**Authors:** Yuan Cheng

**Affiliations:** ^1^ HuaShan Hospital, Fudan University, Shanghai, China, Shanghai China

## Abstract

**Background:**

To investigate the physiological clearance of circulating Aβ by the liver and its role in the pathogenesis of Alzheimer’s disease (AD).

**Method:**

Immunostaining, near‐infrared imaging, and flow cytometry were used to explore the physiological clearance of Aβ by the liver and the impact of aging on Aβ clearance. Liver‐specific LRP‐1 knockdown and functional LRP‐1 minigene (mLRP‐1) expression in mice with AD were used to explore the effects of hepatic Aβ clearance on AD pathogenesis and treatment.

**Result:**

Aβ was detected in hepatocytes of both humans and APP/PS1 mice, and Aβ40 and Aβ42 levels in the outflow blood of the liver (hepatic vein) were 13.9% and 8.9% lower than those in the inflow blood (composites of hepatic artery and portal vein), respectively, suggesting that circulating Aβ is removed when flowing through the liver. The hepatic Aβ uptake was lower in aged mice than in young mice. In addition, specific knockdown of the LRP‐1 gene in hepatocytes reduced hepatic Aβ clearance and increased Aβ levels in both plasma and interstitial fluid in the brain, as well as AD‐type pathologies and behavioral deficits in APP/PS1 mice. Furthermore, enhancing liver Aβ clearance by expressing functional mLRP‐1 in hepatocytes reduced brain Aβ deposition, ameliorated AD‐type pathologies, including neuroinflammation, neuronal loss, and tau hyperphosphorylation, and improved behavioral performance.

**Conclusion:**

Our findings demonstrate that the liver physiologically clears brain‐derived Aβ, providing novel insights into the disease pathogenesis of AD and suggesting new interventional strategies.

